# Reservoir of neurons with adaptive time constants: a hybrid model for robust motor-sensory temporal processing

**DOI:** 10.1186/1471-2202-15-S1-P9

**Published:** 2014-07-21

**Authors:** Sakyasingha Dasgupta, Poramate Manoonpong, Florentin Wörgötter

**Affiliations:** 1 III. Institute for Physics – Biophysics, Georg-August University, Göttingen, Germany; 2 Bernstein Center for Computational Neuroscience, Göttingen, Germany; 3 Mærsk Mc-Kinney Møller Institute, University of Southern Denmark, Odense, Denmark

## 

**Figure 1:**
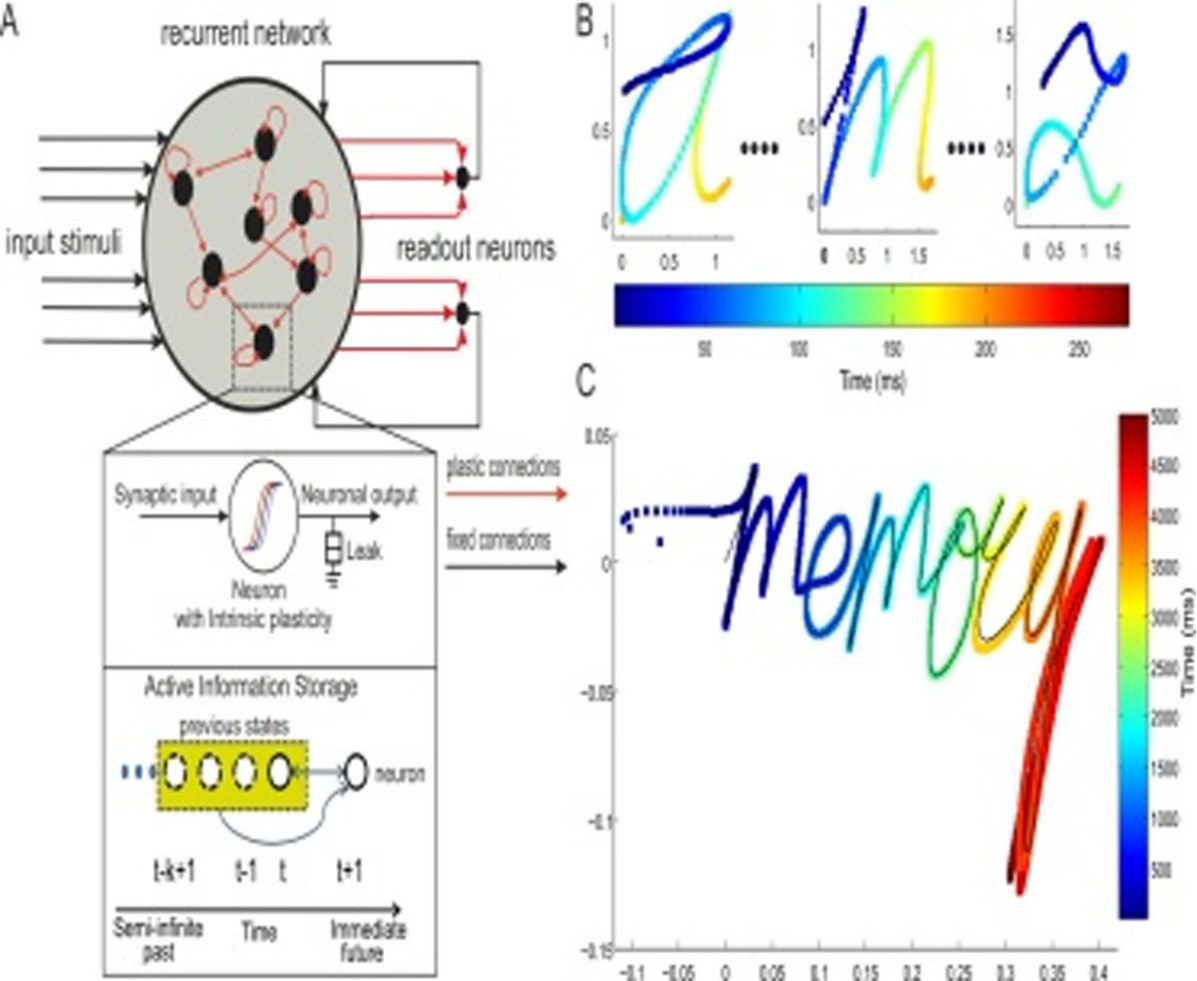
Interval timing dependent handwriting generation task using a reservoir of 3000 rate-coded neurons. A. Basic structure of the recurrent neural network with input, reservoir and output connections for a temporal pattern generation task (inset shows single reservoir neuron properties). B. Inputs to the reservoir consisting of 26x2 dimensions (x and y co-ordinates of alphabets a-z) and an additional auxiliary input fixed at 1.0 (not shown). Inputs are given as a brief stimulus of 210ms. C. Network learns to write the word ‘memory’ cursively. The desired trajectory is shown in black and color coding indicates the exact interval of time when each letter is generated. Intervals from 500ms up to 5000 ms are successfully learned.

The ability to precisely quantify time on the scale of hundreds of milliseconds is critical towards the processing of complex sensory and motor patterns. However, the natures of neural mechanisms for temporal processing (at this scale) in the brain are mostly unknown. Based on experimental data (psychophysics, cell cultures, electrophysiology) and theoretical studies, it is largely debated whether *dedicated* circuits or *intrinsic* mechanisms of neural circuits underlie the timing process [1]. One specific type of timing model, namely state-dependent networks (SDN) [2], shows that time is encoded in the temporal patterns of activity of neural populations and emerges from the internal dynamics of recurrent networks. This can be achieved without the need of dedicated timing units. However, such intrinsic models in their present form have difficulty accounting for crossmodal transfer [1]. In contrast, recent experimental evidence indicates that medial premotor cortical neurons of behaving monkeys show specific interval tuning across modalities (auditory and visual) [3]. In this work we propose a *hybrid* model, making the hypothesis that dedicated interval tuning mechanisms of individual neurons augment the intrinsic dynamics of large recurrent networks (dynamic reservoir). Using a network model of rate-coded neurons starting with random initialization of synaptic connections, we propose a learning rule based on local active information storage (LAIS) [4] to adapt neuronal time constants with respect to the input stimuli to the network. Measured at each spatiotemporal location of the reservoir, LAIS gives a probabilistic measure of the amount of information in the previous state of the neuron that is relevant in predicting the next state. Interestingly high LAIS regions in the network correlate to significant events in time (intervals) of the driving stimulus. Furthermore, we combine this with mutual information driven intrinsic plasticity scheme in order to stabilize chaotic activity in the network. Incoming input drives the network which, in turn, is connected to readout neurons (Figure 1.A) that display the learned behavior for temporally dependent sensory motor tasks. Reservoir-to-output connections can be adapted using both supervised and reward modulated learning rules. Using single and multiple interval discrimination tasks, we show that our network reproduces (across modalities) a linear increase in temporal variability with increase in interval duration. This correlation is also observed in experimental data [3]. Furthermore we demonstrate that our dedicated timing mechanism complements the inherent transient dynamics of the network by successfully learning complex time dependent motor behaviors; like handwriting generation (Figure 1.B and C), locomotion pattern transformation and temporal memory tasks. In essence, our hybrid model demonstrates that time can be encoded by a combination of dedicated and intrinsic mechanisms with the possibility to ‘learn’ the temporal structure of incoming stimuli [5].
